# Cost-effectiveness analysis of sintilimab vs. placebo in combination with chemotherapy as first-line therapy for local advanced or metastatic oesophageal squamous cell carcinoma

**DOI:** 10.3389/fonc.2022.953671

**Published:** 2022-12-06

**Authors:** Taihang Shao, Mingye Zhao, Wenxi Tang

**Affiliations:** ^1^ Center for Pharmacoeconomics and Outcomes Research, China Pharmaceutical University, Nanjing, China; ^2^ Department of Public Affairs Management, School of International Pharmaceutical Business, China Pharmaceutical University, Nanjing, China

**Keywords:** cost-effectiveness analysis, sintilimab, local advanced or metastatic oesophageal squamous cell carcinoma, Orient 15, China

## Abstract

**Objective:**

Results of Orient 15 indicated the health benefits to patients with local advanced or metastatic oesophageal squamous cell carcinoma (OSCC). This study aimed to evaluate the cost-effectiveness of sintilimab plus chemotherapy in treating OSCC from the perspective of Chinese healthcare system.

**Methods:**

A partitioned survival model was constructed to evaluate the cost-effectiveness of sintilimab plus chemotherapy vs. chemotherapy in treating OSCC. Baseline characteristics of patients and key clinical data were extracted from Orient 15. Costs and utilities were collected from published studies and open-access databases. Costs, quality-adjusted life-years (QALYs), life-years gained, and incremental cost-effectiveness ratios (ICER) were chosen as economic outcome indicators. We also performed sensitivity analyses and subgroup analyses to verify the stability of results.

**Results:**

Combination therapy provided additional 0.84 QALYs and 1.46 life-years with an incremental cost of $25,565.48 than chemotherapy, which had an ICER of $30,409.44 per QALY. The probabilistic sensitivity analysis indicated that combination therapy had a 98.8% probability of cost-effectiveness at the willingness-to-pay threshold (WTP) of $38,184 per QALY. Deterministic sensitivity analysis showed that model outcomes were sensitive to the utilities of progression-free survival and progression disease. The subgroup analysis revealed that combination therapy was cost-effective in patients with high expression of PD-L1 and several specific subgroups.

**Conclusion:**

In this economic evaluation, sintilimab plus chemotherapy was likely to be cost-effective compared with chemotherapy in the first-line therapy of advanced OSCC from the perspective of Chinese healthcare system. Our findings may provide evidence for clinicians to make optimal decisions in clinical practice and for decision-makers to evaluate the cost-effectiveness of sintilimab.

## Introduction

Oesophageal cancer ranks seventh in terms of incidence and sixth in mortality worldwide in 2020 (1). Oesophageal squamous cell carcinoma (OSCC) is the predominant subtype of oesophageal cancer in Global populations, with an incidence of 85% (2). And more than half of the number of patients with OSCC are from China (3, 4). For decades, standard first-line treatment for patients with advanced or metastatic OSCC is still limited to platinum-based chemotherapy in China, with a median of 7.0 to 13.0 months based on data from several prospective clinical studies (5–8). Therefore, an effective first-line treatment to improve clinical therapy in these populations is needed.

The emergence of immune checkpoint inhibitors has drastically altered the landscape of cancer treatment. Sintilimab, a fully recombinant human IgG4 anti-PD-1 monoclonal antibody, showed clinical efficacy in many cancers. Sintilimab was also approved by the National Medical Products Administration of China in the treatment of classical Hodgkin’s lymphoma, non-small cell lung cancer, and hepatocellular carcinoma (9–12).

Recently, the randomized, double-blinded, phase III trial Orient 15 reported the efficacy and safety of sintilimab versus placebo in combination with chemotherapy (cisplatin plus paclitaxel or cisplatin plus 5-fluorouracil) as the first-line treatment of unresectable locally advanced, recurrent, or metastatic OSCC (13). The results revealed that sintilimab plus chemotherapy markedly prolonged the median progression-free survival (PFS) in comparison with placebo plus chemotherapy (7.2 months vs. 5.7 months, HR= 0.56, 95% confidence interval 0.46 to 0.68), and greater OS was observed (16.7 months vs. 12.5 months; HR= 0.63, 95% confidence interval 0.51 to 0.78). Thus, sintilimab plus chemotherapy seemed to be an attractive alternative for treating advanced OSCC. The price of sintilimab in the National Reimbursement Drug List (NRDL) is $169.79/100mg. Comprehensively considering the clinical benefits and potential cost-effectiveness, the Chinese government approved this combination therapy for the first-line treatment of OSCC in 2022 (14).

Despite these encouraging clinical results, evidence of cost-effectiveness should not be ignored since the combination therapy had a relatively higher cost than chemotherapy alone. Therefore, this study aimed to investigate the cost-effectiveness of sintilimab plus chemotherapy as the first-line treatment for unresectable locally advanced, recurrent, or metastatic OSCC from the perspective of Chinese healthcare system. The evidence may provide information for clinicians and decision-makers to allocate resources reasonably in health decisions.

## Methods

### Patients and intervention

We reported this study followed the Consolidated Health Economic Evaluation Reporting Standards (CHEERS) reporting guideline (15). Targeted patients were ≥18 years of age with histologically confirmed unresectable locally advanced, recurrent, or metastatic OSCC. Other key characteristics were the same with the Orient 15 trial (13).

Included patients received sintilimab or placebo intravenously at a dose of 3 mg/kg in patients weighing <60 kg or 200 mg in patients weighing ≥60 kg every three weeks (one cycle). Chemotherapy included cisplatin (75 mg/m^2^ every cycle) plus paclitaxel (175 mg/m^2^ every cycle) or 5-fluorouracil (800 mg/m^2^ continuous administration for five days in each cycle). A maximum of six cycles was recommended for chemotherapy. When disease progressed, or intolerable toxicity occurred, patients could alter from the first-line treatments to the second-line treatments. In Orient 15, 41% (134/327) in sintilimab plus chemotherapy group and 54% (179/332) in placebo plus chemotherapy group received subsequent therapies (13).

### Model structure

We constructed a partitioned survival model with three health states in our economic evaluation (16). The three health states were progression-free survival (PFS), progressed disease (PD), and death (D). We set the time horizon as 10 years to allow 99% of patients dying in both treatment arms. The cycle length was set as 21 days according to the original research cycle. The analysis was conducted from the perspective of Chinese healthcare system. The primary outputs of the model were cost, quality-adjusted life-years (QALYs), life-years gained, and incremental cost-effectiveness ratio (ICER). Both costs and clinical outcomes were discounted by 5% annually (17). Willingness-to-pay (WTP) threshold was set as $38,184 (three times GDP per capita) per QALY gained (18). The model was constructed using R 4.1.2 (https://www.r-project.org/) and Microsoft Excel 2019 (Redmond, Washington, United States).

### Clinical data input

Since individual patient data (IPD) was unavailable, we extracted the PFS and OS data from the Kaplan-Meier (KM) curves of the Orient 15 trial. Then, we used the method of Guyot et.al (19). to reconstruct the IPD over the follow-up time of the trial through GetData Graph Digitizer (http://getdata-graph-digitizer.com). We precisely reproduced the digitized KM curves since the reconstructed patient-level data whose event and censor times were equal in number to the initial number at risk. Parametric models were applied to extrapolate the survival data to the long term. Following parametric survival models were considered: exponential, Weibull, Gompertz, gamma, log-logistic, log-normal, generalized gamma, genf, fractional polynomial (FP), restricted cubic spline models (RCS) and Royston-Parmar models (RP) (20–23). The eligible parametric survival model was chosen based on the lowest value of the Akaike information criterion and visual inspection (20, 24). The final parametric survival models of the two treatments are shown in **Table 1**, and the goodness-of-fit results are shown in **Supplementary Tables 1, 2**. The validation plot and survival distribution are shown in **Supplementary Figures 1–12**. Data of subsequent treatment was collected from Orient 15. The clinical data inputs in detail are given in **Table 1**.

**Table 1:** Key model parameters input.

Parameters	Value	Range (Upper, Lower)	Distribution	Source
**Cost**				
Cost of sintilimab (100mg)	169.79	(135.83, 169.79)	Gamma	(25, 26)
Cost of Cisplatin per unit (10mg)	1.47	(1.38, 1.47)	Gamma	
Cost of Cisplatin per unit (30mg)	3.01	(3.01, 4.4)	Gamma	
Cost of paclitaxel per unit (100mg)	27.98	(27, 28)	Gamma	
Cost of 5-fluorouracil	9.12	(7.29, 10.94)	Gamma	
Cost of Camrelizumab	460.31	(368.25, 552.37)	Gamma	
Cost of Anlotinib	675.42	(540.34, 675.42)	Gamma	
Cost of Docetaxel	24.87	(24.76, 59.56)	Gamma	
Cost of BSC	116.14	(92.91, 139.36)	Gamma	(27)
Routine follow-up cost per cycle	51.07	(40.86, 61.29)	Gamma	
Cost of laboratory tests and radiological examinations	247.56	(198.05, 297.07)	Gamma	
Cost of salvage therapy per cycle	443.21	(354.57, 531.85)	Gamma	
Cost of end-of-life	1,460.30	(1,168.24, 1,752.36)	Gamma	
Cost of neutrophil count decreased	116.37	(51.11, 357.8)	Gamma	(25, 26, 28)
Cost of white blood cell count decreased	116.37	(51.11, 357.8)	Gamma	
Cost of lymphocyte count decreased	116.37	(51.11, 357.8)	Gamma	
Cost of platelet count decreased	1,523.82	(1,240.17, 1,771.67)	Gamma	
Cost of anemia	140.40	(106.73, 160.1)	Gamma	
Cost of pneumonia	1,640.00	(1,312, 1,968)	Gamma	(29)
Cost of increase in blood pressure	113.53	(106.73, 160.1)	Gamma	(30)
Cost of hypokalaemia	3,223.00	(2,578.4, 3,867.6)	Gamma	(31)
Cost of asthenia	107.00	(80, 134)	Gamma	(32)
**Utility**				
Utility of PFS	0.75	(0.6, 0.9)	Beta	(33)
Utility of PD	0.67	(0.54, 0.8)	Beta	
Disutility of neutrophil count decreased	0.20	(0.16, 0.24)	Beta	(34)
Disutility of lymphocyte count decreased	0.20	(0.16, 0.24)	Beta	(28)
Disutility of white blood cell count decreased	0.20	(0.16, 0.24)	Beta	(34)
Disutility of platelet count decreased	0.11	(0.09, 0.13)	Beta	(35)
Disutility of anemia	0.07	(0.06, 0.09)	Beta	(36)
Disutility of pneumonia	0.05	(0.04, 0.06)	Beta	(37)
Disutility of increase in blood pressure	0.08	(0.06, 0.1)	Beta	(34)
Disutility of hypokalaemia	0.03	(0.02, 0.04)	Beta	(31)
Disutility of asthenia	0.10	(0.09, 0.11)	Beta	(38)
**Risk of adverse events (≥ grade 3)**				
**Sintilimab Group**				
Neutrophil count decreased	0.30	(0.24, 0.36)	Beta	(13)
White blood cell count decreased	0.17	(0.14, 0.21)	Beta	
Platelet count decreased	0.03	(0.02, 0.03)	Beta	
Anemia	0.13	(0.1, 0.15)	Beta	
Pneumonia	0.03	(0.02, 0.04)	Beta	
Hypokalaemia	0.05	(0.04, 0.06)	Beta	
Increase in blood pressure	0.03	(0.02, 0.04)	Beta	
**Chemotherapy Group**				
Neutrophil count decreased	0.34	(0.27, 0.41)	Beta	(13)
White blood cell count decreased	0.22	(0.18, 0.27)	Beta	
Platelet count decreased	0.03	(0.02, 0.04)	Beta	
Anemia	0.10	(0.08, 0.12)	Beta	
Asthenia	0.04	(0.03, 0.04)	Beta	
Lymphocyte count decreased	0.04	(0.03, 0.05)	Beta	
**Other Parameters**				
Discount rate	0.05	(0, 0.08)	Beta	(17)
Weight	60.00	(48, 72)	Gamma	(13)
Proportion of paclitaxel used in Sintilimab Group	0.91	(0.73, 1)	Beta	
Proportion of paclitaxel used in Chemotherapy Group	0.93	(0.75, 1)	Beta	
**Proportion of Subsequent treatment**				
**Sintilimab Group**				
Proportion of Subsequent treatment_Immountherapy	0.13	(0.1, 0.16)	Beta	(13)
Proportion of Subsequent treatment_Targeted drugs	0.08	(0.06, 0.1)	Beta	
Proportion of Subsequent treatment_chemotherapy	0.20	(0.16, 0.24)	Beta	
Proportion of Subsequent treatment_BSC	0.59	(0.67, 0.51)	Beta	
**Chemotherapy Group**				
Proportion of Subsequent treatment_Immountherapy	0.18	(0.14, 0.22)	Beta	(13)
Proportion of Subsequent treatment_Targeted drugs	0.12	(0.1, 0.14)	Beta	
Proportion of Subsequent treatment_chemotherapy	0.23	(0.18, 0.28)	Beta	
Proportion of Subsequent treatment_BSC	0.47	(0.58, 0.36)	Beta	

PD, progressed disease; PFS, progression-free survival; BSC, best-supportive care.

All costs were calculated in USD.

### Cost and utility input

In our study, only direct medical costs were considered, including costs of acquiring drugs, costs attributed to the patient’s diagnosis and hospitalization, costs for the management of adverse events (AEs), and costs for end-of-life care (Eol) were analyzed. Drug prices were obtained from public databases and were up to date in 2021 (25, 26). We exchanged the prices in RMB to US$ with the exchange rate of 6.36 (Feb 7, 2022). Since cisplatin and paclitaxel had multiple dosage forms in Chinese market, we chose the most reasonable dosage combination to meet the balance of both effect and lower cost (39). We extracted mean body weight from Orient 15 to calculate drugs administered based on patients’ weight (13). We only considered severe AEs (≥grade 3) with rates over 3%, including anemia, pneumonia, hypokalaemia, and five other AEs. For subsequent treatment, we considered only camrelizumab for immunotherapy, anlotinib for targeted drugs, and docetaxel for chemotherapy based on the current Chinese clinical guideline (40). All cost-related parameters are shown in **Table 1**.

The utilities of PFS and PD states associated with advanced OSCC were 0.75 and 0.67 respectively, which were derived from a cost-effectiveness analysis based on the E-DIS trial (33). The disutility values due to AEs were included in this analysis and were extracted from other studies (28). All AEs were assumed to be incurred during the first cycle (41). The duration-adjusted disutility was subtracted from the baseline PFS utility. All utility-related parameters are shown in **Table 1**.

### Sensitivity analysis and subgroup analysis

Sensitivity analyses were conducted to test the robustness of the model. In deterministic sensitivity analysis (DSA), all parameters were adjusted within the reported 95% confidence intervals (CI), which were derived from the previous studies or assuming reasonable ranges of the base case values ( ± 20%). We conducted the probabilistic sensitivity analysis (PSA) through a Monte Carlo simulation of 10,000 iterations. We selected a gamma distribution for cost and a beta distribution for probability, proportion, and utility. We used the scatter plot and cost-effectiveness acceptability curves (CEAC) to analyze the cost-effectiveness for each treatment with different WTP thresholds.

In subgroup analysis, we first analyzed the results for patients with combined positive scores (CPS) of ≥10 for expression of PD-L1 through the methods of base case analysis since the Orient 15 reported the independent KM curves for these patients. For other subgroups, the ICERs were calculated using the subgroup-specific HRs for OS and PFS obtained from Orient 15. We considered the subgroup of patients with different sex, age, baseline weight, country or region, Eastern Cooperative Oncology Group (ECOG) performance status score, disease type, Hepatic metastasis, and chemotherapy. Data for all subgroups except for the HRs for OS and PFS was assumed to be the same since the lack of sufficient data. Therefore, proportional hazards were assumed here. The composition of costs would be calculated based on specific subgroups.

## Results

### Base-case analysis results

Results of cost, utilities, and life-years gained are shown in **Table 2**. Compared with chemotherapy alone, sintilimab plus chemotherapy showed an additional 1.459 life-years in OS, with an increased QALYs of 0.841. The ICER of sintilimab plus chemotherapy compared with chemotherapy was $30,409.44/QALY. When only focused on PFS period, sintilimab plus chemotherapy also exhibited an additional life-years of 0.612 and increased QALYs of 0.418.

**Table 2:** Results of base-case analysis and scenario analysis.

Drug	OS			Only PFS			Total		
	Cost(95%CI)	Life-years	Utility(95%CI)	Cost(95%CI)	Life-years	Utility(95%CI)	Increment cost(95%CI)	Increment utility(95%CI)	ICER(95%CI)
base-case analysis									
Chemotherapy	18,071.13 (18,055.61-18,093.51)	1.323	0.871 (0.870-0.872)	9,177.51 (9,169.85-9,189.65)	0.726	0.482 (0.481-0.483)	——	——	——
Sintilimab plus chemotherapy	43,636.61 (43,603.14-43,679.08)	2.782	1.712 (1.710-1.714)	22,691.80 (22,673.36-22,711.76)	1.338	0.900 (0.898-0.901)	25,565.48 (25,546.61-25,586.48)	0.841 (0.840-0.842)	30,409.44 (30,343.93-30,534.52)
scenario analysis									
Chemotherapy	20,783.76 (20,741.69-20,797.99)	1.522	1.002 (1.001-1.004)	11,606.04 (11,598.02-11,614.03)	0.904	0.598 (0.596-0.599)	——	——	——
Sintilimab plus chemotherapy	40,065.40 (39,999.90-40,089.46)	2.523	1.598 (1.595-1.600)	30,775.25 (30,724.66-30,794.23)	2.005	1.267 (1.264-1.270)	19,281.64 (19,256.47-19,293.21)	0.595 (0.594-0.597)	32,376.78 (32,277.35-32,497.35)

ICER, incremental cost-effectiveness ratio; QALY, quality-adjusted life years; PFS, progression-free survival; OS, overall survival; CI, confidence interval.

Unit of cost is USD; unit of utility is QALY.

95% confidence intervals of the life-years are not provided, since the input survival rate is fixed.

### Sensitivity analysis

**Figure 1:**
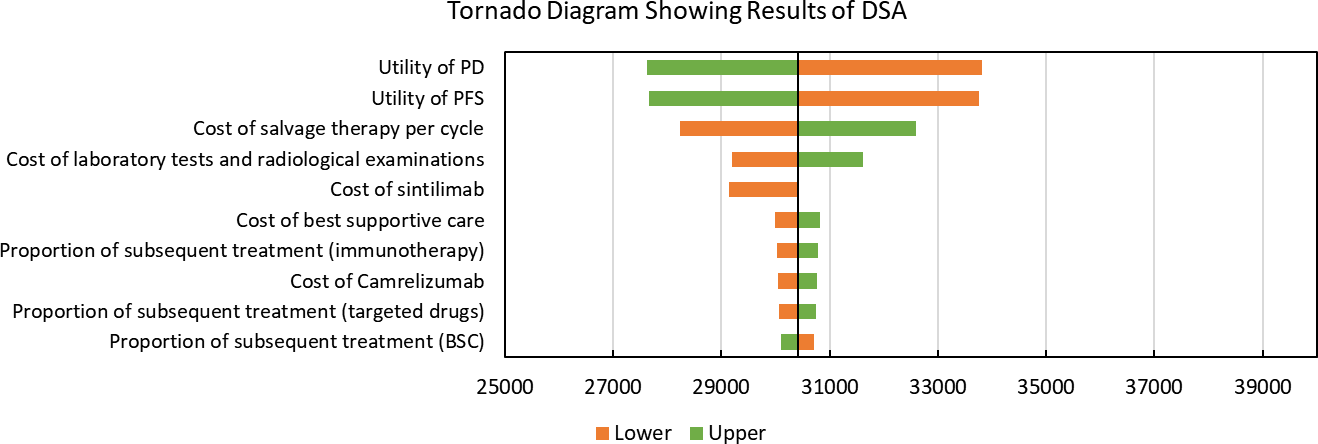
Tornado Diagram Showing Results of Deterministic Sensitivity Analysis. DSA, Deterministic Sensitivity Analysis; PD, progressed disease; PFS, progression-free survival; BSC, best-supportive care; QALY: quality-adjusted life years. The vertical black line represents the primary result of $30409.44 per QALY.

Results of DSA are shown in **Figure 1**. Utilities of PFS and PD were the most significantly factors that associated with model outcomes. Cost of salvage therapy, sintilimub, laboratory tests and radiological examinations also significantly impacted the ICERs. Other parameters like subsequent treatment proportions had only moderate or low associations with the model outcomes. In addition, with all parameters fluctuating in the upper and lower limits, the modeled ICERs did not exceed the given WTP threshold.

**Figure 2:**
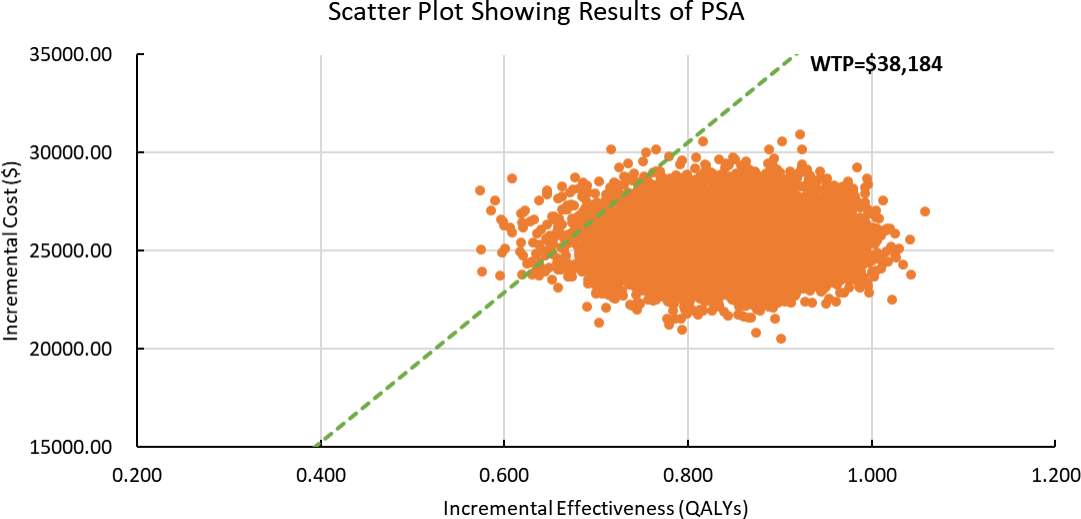
Scatter Plot Showing Results of Probabilistic Sensitivity Analysis. PSA, Probabilistic Sensitivity Analysis; WTP, willingness-to-pay; QALY, quality-adjusted life years. The dashed green line represents the WTP thresholds; the orange scatter points represent the simulated ICER points.

**Figure 3:**
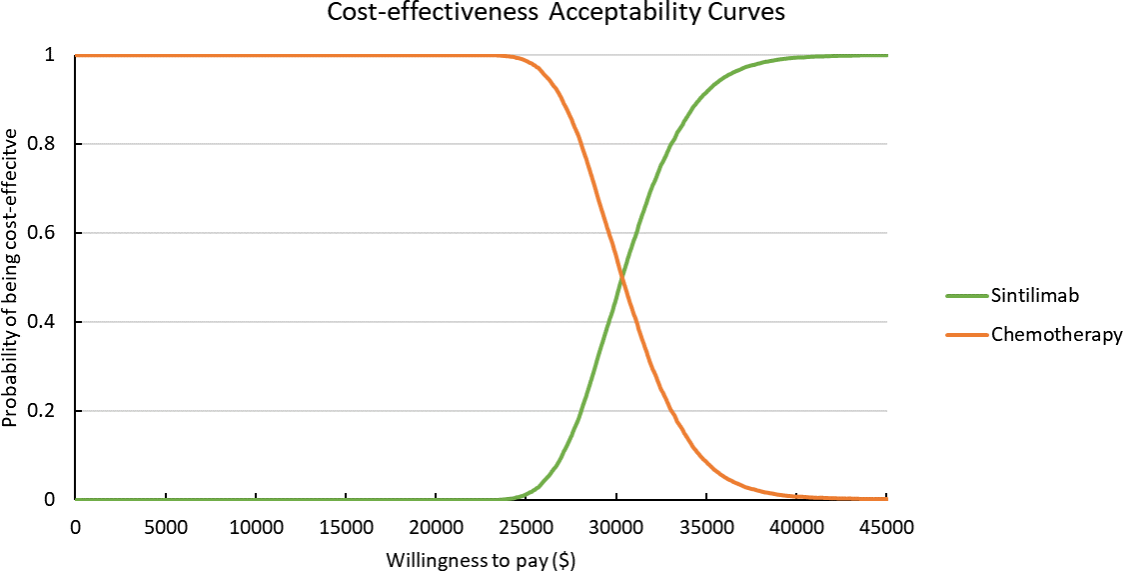
Cost-effectiveness Acceptability Curves. Green line represents the sintilimab group; orange line represents the chemotherapy group.

Results of PSA are shown in **Figure 2**. According to the scatter plot, when WTP was set as three times GDP per capita, the probabilities that combination therapy being cost-effective reached 98.8%. Cost-effectiveness acceptability curve showed that when the WTP threshold was set smaller than $30,300, chemotherapy was more likely to be cost-effective. When the WTP threshold exceeded $30,300, sintilimab plus chemotherapy began to obtain a more than 50% probability of being cost-effective (**Figure 3**).

### Subgroup analysis

In the first subgroup, we considered comparing the cost-effectiveness of two therapies among patients whose CPS ≥10. Results of the scenario analysis were similar to the results of base case analysis, whose ICER was $32,376.78/QALY (**Table 2**). Sintilimab plus chemotherapy was associated with an additional 1.001 life-years gained and 0.596 increased QALYs than chemotherapy alone.

**Figure 4:**
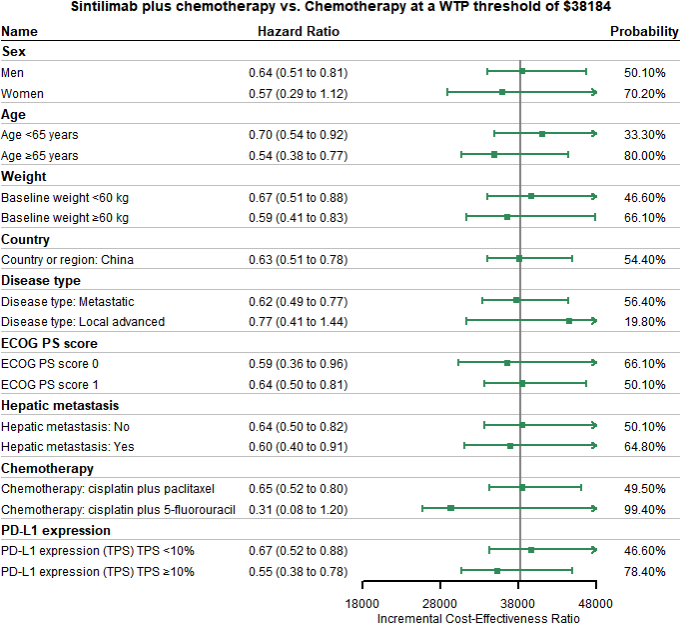
Subgroup Analysis Results of Incremental Cost-effectiveness Ratios (ICERs) and Probabilities of Cost-effectiveness Obtained by Varying the Hazard Ratios (HRs) for Overall Survival. ECOG PS, Eastern Cooperative Oncology Group performance status score; TPS, tumor cell proportion score.

Assumed HR to be constant, we conducted the subgroup analysis for other subgroups like sex, age, and weight for both OS and PFS. ICERs changed with HR were selected as the outcome indicator, and the probability of being cost-effective for sintilimab plus chemotherapy was also calculated. Summary results of subgroup analysis are concluded in **Figure 4** and **Supplementary Figure 13**. The subgroup analysis performed by varying the HRs for PFS found that sintilimab plus chemotherapy was associated with the probability of being cost-effective was higher than 50% in all subgroups. The subgroup analysis performed by varying the HRs for OS revealed that sintilimab plus chemotherapy in the following subgroups still had a probability of more than 50% to be cost-effective with the given WTP threshold: both men and women, patients with age ≥ 65 years (probability of being cost-effective, 80%), patients with baseline weight ≥60kg (probability of being cost-effective, 66.1%), patients from China (probability of being cost-effective, 54.4%), patients whose disease type were metastatic (probability of being cost-effective, 56.4%), patients whose ECOG PS score equal to both 1 and 0, patients with or without hepatic metastasis, patients who use the chemotherapy of cisplatin plus 5-fluorouracil (probability of being cost-effective, 99.4%) and patients with PD-L1 expression TPS ≥10% (probability of being cost-effective, 78.4%).

## Discussion

Our study is the first to conduct the economic evaluation of sintilimab plus chemotherapy versus chemotherapy alone in patients with unresectable locally advanced, recurrent, or metastatic OSCC. Based on the results of Orient 15, our base-case analysis showed that sintilimab plus chemotherapy had additional life-years gained and increased QALYs than chemotherapy. Combination therapy was also more cost-effective for OSCC under the WTP threshold of $38,184 per QALY. Base-case analysis results were robust according to the results of DSA and PSA. According to our DSA results, utilities of PFS and PD, cost of salvage therapy, sintilimub, laboratory tests and radiological examinations were significantly associated with the model outcomes. In the subgroup of patients with CPS ≥10, sintilimab plus chemotherapy also exhibited cost-effectiveness under the given WTP threshold. 12 subgroups, including sex (men or women), age ≥ 65 years, baseline weight ≥60kg, region China, disease type of metastatic, ECOG PS score, hepatic metastasis, the chemotherapy of cisplatin plus 5-fluorouracil and PD-L1 expression TPS showed that sintilimab plus chemotherapy had a higher than 50% probability of cost-effectiveness compared with chemotherapy.

According to DSA, utilities of PFS and PD were two factors with the most significant influences. Utilities used in this study were extracted from a published economic evaluation study that targeted on the cost-effectiveness of continuation versus discontinuation of first-line chemotherapy in patients with metastatic OSCC. Some biases might occur since patients included in this study were not Chinese. However, our DSA results showed that with utilities fluctuating, ICERs were still below the given WTP threshold, which indicated that the fluctuation of these two factors did not affect the conclusion.

Patients with CPS ≥10 were a key subgroup in Orient 15. Significant improved clinical benefits were observed in this subgroup for both PFS and OS. Similar results were found in other clinical trials: Keynote-590, Checkmate-648, and ESCORT-1st, which indicated that patients with higher expression of PD-L1 derive more clinical benefit (42–44). Subgroup analysis results revealed that for patients with either PD-L1 expression of CPS ≥ 10 or PD-L1 expression of TPS ≥ 10%, sintilimab plus chemotherapy could achieve cost-effectiveness. Therefore, sintilimab plus chemotherapy should be considered as a choice for advanced OSCC patients with higher expression of PD-L1.

The ability of sintilimab plus chemotherapy to improve the PFS and OS benefits over chemotherapy was associated with economic outcomes. Our base-case analysis found that combination therapy provided patients with more clinical benefits in both PFS and OS. By assuming HR to be constant, the cost-effectiveness of each subgroup could be calculated. We found that for PFS, the fluctuation of HRs did not have significant impacts on conclusions. However, for OS, the fluctuation of HRs in some groups could affect the results. This indicated that for specific subgroups, sintilimab plus chemotherapy was still cost-effective. This finding might provide evidence for clinicians to make optimal decisions with limited healthcare resources.

Currently, few studies are targeted on the economic evaluations of immunotherapies for advanced OSCC. After a systematic search on PubMed, nine CEA studies were found to assess different immunotherapies for advanced OSCC. Seven of them focused on second-line therapies. One of the remaining two focused on the cost-effectiveness of first-line camrelizumab plus chemotherapy for advanced or metastatic OSCC in China from the perspective of the Chinese healthcare system (27). Camrelizumab plus chemotherapy was estimated not cost-effective according to this study. Another study analyzed the cost-effectiveness of first-line pembrolizumab plus 5-fluorouracil and cisplatin for advanced or metastatic OSCC from the perspective of USA and China. The study indicated that pembrolizumab combination therapy was not cost-effective (45). Therefore, our study firstly evaluated the cost-effectiveness of sintilimab plus chemotherapy which could provide more evidence for clinicians about sintilimab in practice. Furthermore, sintilimab has been included in National Reimbursement Drug List (NRDL) since 2019 (46). Currently, four indications of sintilimab have been listed in NRDL, including non-squamous non-small cell lung cancer, squamous non-small cell lung cancer, hepatic cancer, and Hodgkin lymphoma. Thus, our findings could also provide evidence for decision-makers to consider the cost-effectiveness of sintilimab for advanced OSCC.

There are several limitations in this study. First, owing to the lack of head-to-head study, we did not include other first-line immunotherapies, such as pembrolizumab, nivolumab, camrelizumab and toripalimab, which have shown favorable health benefits for patients with advanced OSCC (42–44, 47–50). Second, this economic evaluation was based on the interim analysis of Orient 15. Therefore, some biases might exist in the survival outcomes, subgroup analysis results and adverse events. Third, partitioned survival model was used in this study. Since this model could not reflect the time-varying transition rate between health states, the estimation of survival outcomes might be different from the actual clinical situation. Therefore, future final analysis results of Orient 15 are needed to validate and improve this study. Forth, clinical benefits beyond the observation time of Orient 15 were predicted by parametric models. This might lead to biases in the model outcomes, although flexible parametric models were applied, and the modeled and observed data were validated. Fifth, costs and disutilities of grade 1 or 2 AEs were excluded, which may result in overestimating the economic outcomes associated with sintilimab plus chemotherapy. This limitation may not influence outcomes significantly, as DSA results indicated that the impacts associated with AEs were minor. Sixth, utilities of PFS and PD in this study were obtained from other trials. According to our DSA results, although utilities had the greatest impacts on the base-case analysis results, the conclusion remained unchanged.

## Conclusions

The findings of this cost-effectiveness analysis suggested that from the perspective of Chinese healthcare system, sintilimab plus chemotherapy is likely to be cost-effective at WTP thresholds of $38,184 per QALY compared with chemotherapy for patients with advanced OSCC. Patients with high expression of PD-L1 and specific subgroups could achieve more clinical benefits and favorable cost-effectiveness from this new combination therapy. Future long-term data are needed to validate and improve this study.

## Data availability statement

The original contributions presented in the study are included in the article/**Supplementary Material**. Further inquiries can be directed to the corresponding author.

## Author contributions

All authors developed the concept and design of the study. TS and MZ performed the data collection, analyzed the data, and prepared the manuscript, and WT performed critical revision of the manuscript. All authors contributed to the article and approved the submitted version.
