# Supplementary material for: Cost-effectiveness analysis of sintilimab vs. placebo in combination with chemotherapy as first-line therapy for local advanced or metastatic oesophageal squamous cell carcinoma
Source: Front Oncol. 2022 Dec 6;12:953671. doi: 10.3389/fonc.2022.953671 (PMC9763586; doi:10.3389/fonc.2022.953671)
Supplement: Supplementary file 1 [file DataSheet_1.docx]

**Supplementary Material**

**Content**

[Supplementary Table 1 2](#_Toc20316)

[Supplementary Table 2 3](#_Toc5978)

[Supplementary Figure 1 4](#_Toc28846)

[Supplementary Figure 2 5](#_Toc6906)

[Supplementary Figure 3 6](#_Toc23209)

[Supplementary Figure 4 7](#_Toc31637)

[Supplementary Figure 5 8](#_Toc26077)

[Supplementary Figure 6 9](#_Toc14605)

[Supplementary Figure 7 10](#_Toc24015)

[Supplementary Figure 8 11](#_Toc22342)

[Supplementary Figure 9 12](#_Toc11161)

[Supplementary Figure 10 13](#_Toc17577)

[Supplementary Figure 11 14](#_Toc28371)

[Supplementary Figure 12 15](#_Toc10742)

[Supplementary Figure 13 16](#_Toc16028)

### Supplementary Table 1

| Supplementary Table 1 Goodness-of-fit results of different parametric models for base-case analysis | | | | | | | | | | | | |
| --- | --- | --- | --- | --- | --- | --- | --- | --- | --- | --- | --- | --- |
|  | Sin-OS | | | Che-OS | | | Sin-PFS | | | Che-OS | | |
| Model | LnL | Params | AIC | LnL | Params | AIC | LnL | Params | AIC | LnL | Params | AIC |
| Exponential | -84.59 | 1 | 171.18 | -93.68 | 1 | 189.37 | -114.36 | 1 | 230.73 | -110.86 | 1 | 223.73 |
| Weibull | -70.91 | 2 | 145.81 | -75.13 | 2 | 154.26 | -101.78 | 2 | 207.55 | -87.18 | 2 | 178.36 |
| Gamma | -69.19 | 2 | 142.39 | ***-74.20*** | ***2*** | ***152.39*** | -96.58 | 2 | 197.16 | -81.88 | 2 | 167.76 |
| Log normal | -70.28 | 2 | 144.56 | -78.97 | 2 | 161.94 | -85.55 | 2 | 175.09 | -81.25 | 2 | 166.50 |
| Gompertz | -78.70 | 2 | 161.40 | -81.75 | 2 | 167.50 | -112.45 | 2 | 228.91 | -103.14 | 2 | 210.27 |
| Log logistic | -67.08 | 2 | 138.16 | -74.69 | 2 | 153.37 | -88.21 | 2 | 180.42 | ***-77.38*** | ***2*** | ***158.76*** |
| Gengamma | -68.41 | 3 | 142.82 | -74.14 | 3 | 154.27 | -84.04 | 3 | 174.08 | -79.19 | 3 | 164.39 |
| GenF | -63.93 | 4 | 135.87 | -74.12 | 4 | 156.24 | -78.55 | 4 | 165.11 | -77.15 | 4 | 162.31 |
| FP1-1 | -69.89 | 2 | 143.78 | -74.25 | 2 | 152.51 | -90.18 | 2 | 184.35 | -83.21 | 2 | 170.42 |
| FP1-2 | -84.85 | 2 | 173.71 | -89.30 | 2 | 182.60 | -91.95 | 2 | 187.89 | -83.67 | 2 | 171.34 |
| FP2-1 | -63.44 | 3 | 132.88 | -73.79 | 3 | 153.58 | -83.65 | 3 | 173.31 | -76.62 | 3 | 159.25 |
| FP2-2 | -63.44 | 3 | 132.88 | -73.85 | 3 | 153.70 | -83.69 | 3 | 173.38 | -76.64 | 3 | 159.28 |
| RCS1 | -63.98 | 3 | 133.96 | -77.15 | 3 | 160.30 | -96.92 | 3 | 199.84 | -81.19 | 3 | 168.37 |
| RCS2 | -63.65 | 4 | 135.29 | -73.62 | 4 | 155.24 | -81.58 | 4 | 171.16 | -80.00 | 4 | 168.00 |
| RP-hazard-1 | -64.21 | 4 | 136.42 | -74.10 | 3 | 154.19 | -74.91 | 7 | 163.82 | -77.70 | 5 | 165.40 |
| RP-hazard-2 | -64.23 | 5 | 138.46 | -75.13 | 2 | 154.26 | -75.69 | 6 | 163.39 | -75.96 | 7 | 165.92 |
| RP-odds-1 | ***-64.14*** | ***4*** | ***136.28*** | -74.69 | 2 | 153.37 | -74.97 | 7 | 163.94 | -77.38 | 2 | 158.76 |
| RP-odds-2 | -67.08 | 2 | 138.16 | -74.43 | 3 | 154.85 | -75.96 | 6 | 163.92 | -77.10 | 3 | 160.21 |
| RP-normal-1 | -64.24 | 4 | 136.47 | -74.22 | 3 | 154.43 | ***-74.51*** | ***7*** | ***163.02*** | -78.43 | 3 | 162.85 |
| RP-normal-2 | -64.04 | 5 | 138.08 | -74.15 | 4 | 156.30 | -75.51 | 6 | 163.03 | -78.06 | 5 | 166.13 |
| Sin: Sintilimab plus chemotherapy; Che: chemotherapy; OS: overall survival; PFS: progression-free survival; LnL: log likelihood; Params: Number of parameters; AIC: Akaike information criterion; FP: fractional polynomial; RCS: restricted cubic spline models; RP: Royston-Parmar models. | | | | | | | | | | | | |

### Supplementary Table 2

| Supplementary Table 2 Goodness-of-fit results of different parametric models for subgroup analysis in patients with PD-L1 expression CPS ≥ 10 | | | | | | | | | | | | |
| --- | --- | --- | --- | --- | --- | --- | --- | --- | --- | --- | --- | --- |
|  | Sin-OS | | | Che-OS | | | Sin-PFS | | | Che-OS | | |
| Model | LnL | Params | AIC | LnL | Params | AIC | LnL | Params | AIC | LnL | Params | AIC |
| exp | -72.86 | 1 | 147.71 | -78.54 | 1 | 159.07 | -89.77 | 1 | 181.54 | -84.38 | 1 | 170.75 |
| weibull | -61.01 | 2 | 126.03 | -66.41 | 2 | 136.83 | -82.23 | 2 | 168.45 | -71.62 | 2 | 147.25 |
| gamma | -59.72 | 2 | 123.44 | -65.32 | 2 | 134.65 | -79.11 | 2 | 162.21 | -66.83 | 2 | 137.66 |
| lnorm | -59.16 | 2 | 122.31 | -65.09 | 2 | 134.18 | -72.34 | 2 | 148.67 | -60.78 | 2 | 125.56 |
| gompertz | -66.69 | 2 | 137.38 | -71.18 | 2 | 146.37 | -88.51 | 2 | 181.03 | -81.97 | 2 | 167.93 |
| llogis | -58.80 | 2 | 121.60 | -65.41 | 2 | 134.82 | -74.56 | 2 | 153.12 | -60.40 | 2 | 124.80 |
| gengamma | -59.00 | 3 | 123.99 | -64.67 | 3 | 135.35 | -70.91 | 3 | 147.82 | -60.70 | 3 | 127.40 |
| genf | -57.22 | 4 | 122.44 | -64.68 | 4 | 137.35 | -67.70 | 4 | 143.39 | -59.95 | 4 | 127.91 |
| FP1-1 | -59.67 | 2 | 123.33 | ***-63.98*** | ***2*** | ***131.96*** | -75.29 | 2 | 154.59 | -63.67 | 2 | 131.35 |
| FP1-2 | -59.70 | 2 | 123.41 | -64.42 | 2 | 132.83 | -76.37 | 2 | 156.74 | -64.44 | 2 | 132.88 |
| FP2-1 | -56.89 | 3 | 119.78 | -63.73 | 3 | 133.47 | -70.96 | 3 | 147.92 | -56.69 | 3 | 119.39 |
| FP2-2 | -56.93 | 3 | 119.87 | -63.75 | 3 | 133.50 | -71.12 | 3 | 148.24 | -57.14 | 3 | 120.29 |
| RCS1 | -57.88 | 3 | 121.75 | -68.34 | 3 | 142.69 | -81.69 | 3 | 169.39 | -60.73 | 3 | 127.46 |
| RCS2 | -57.88 | 4 | 123.77 | -66.15 | 4 | 140.29 | -67.47 | 4 | 142.94 | -60.35 | 4 | 128.69 |
| RP-hazard-1 | -58.13 | 4 | 124.27 | -64.78 | 3 | 135.55 | -65.17 | 7 | 144.34 | -57.40 | 5 | 124.81 |
| RP-hazard-2 | -57.24 | 6 | 126.48 | -66.41 | 2 | 136.83 | -68.45 | 3 | 142.91 | -56.59 | 6 | 125.19 |
| RP-odds-1 | ***-58.80*** | ***2*** | ***121.60*** | -65.41 | 2 | 134.83 | -65.55 | 7 | 145.10 | -60.40 | 2 | 124.80 |
| RP-odds-2 | -56.51 | 6 | 125.02 | -65.36 | 3 | 136.73 | ***-68.45*** | ***3*** | ***142.89*** | -57.57 | 5 | 125.15 |
| RP-normal-1 | -56.24 | 6 | 124.48 | -65.09 | 2 | 134.18 | -65.45 | 7 | 144.90 | ***-57.00*** | ***5*** | ***123.99*** |
| RP-normal-2 | -59.16 | 2 | 122.31 | -64.67 | 3 | 135.35 | -68.07 | 4 | 144.14 | -55.11 | 7 | 124.23 |
| Sin: Sintilimab plus chemotherapy; Che: chemotherapy; OS: overall survival; PFS: progression-free survival; LnL: log likelihood; Params: Number of parameters; AIC: Akaike information criterion; FP: fractional polynomial; RCS: restricted cubic spline models; RP: Royston-Parmar models. | | | | | | | | | | | | |

### Supplementary Figure 1

**
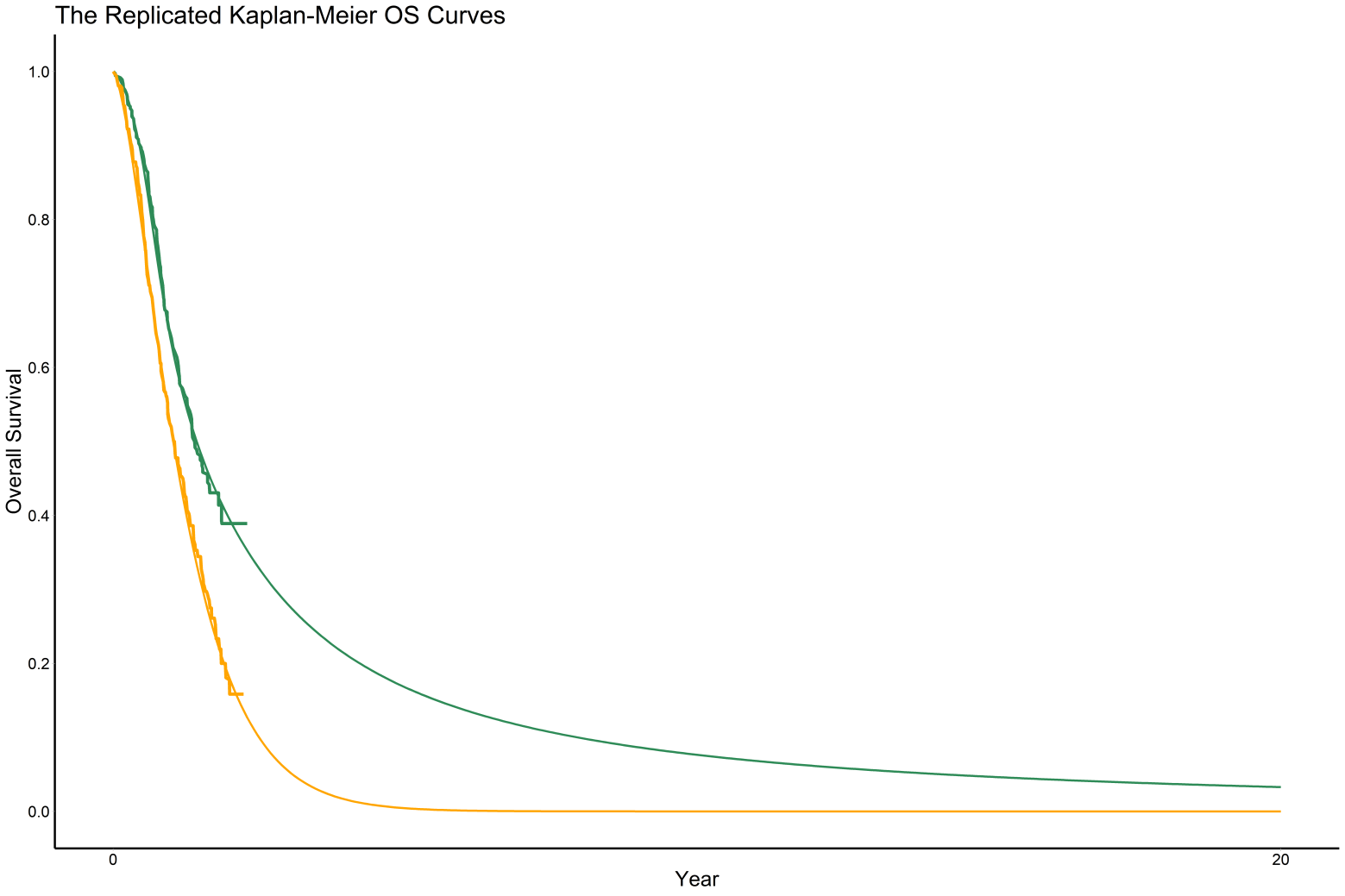
**

**Supplementary Figure 1 The Replicated Kaplan-Meier OS Curves**

### Supplementary Figure 2

**
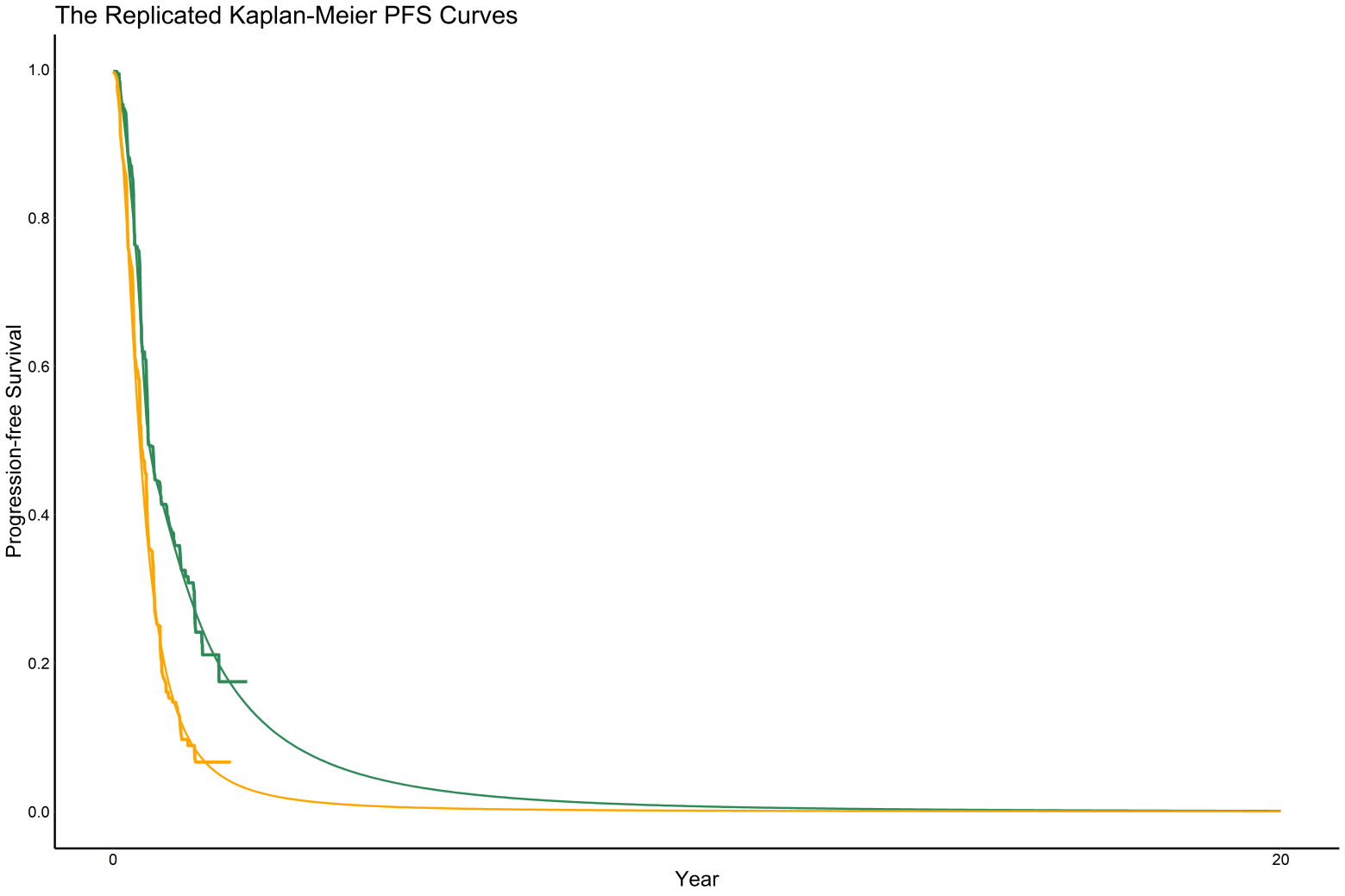
**

**Supplementary Figure 2 The Replicated Kaplan-Meier PFS Curves**

### Supplementary Figure 3

**
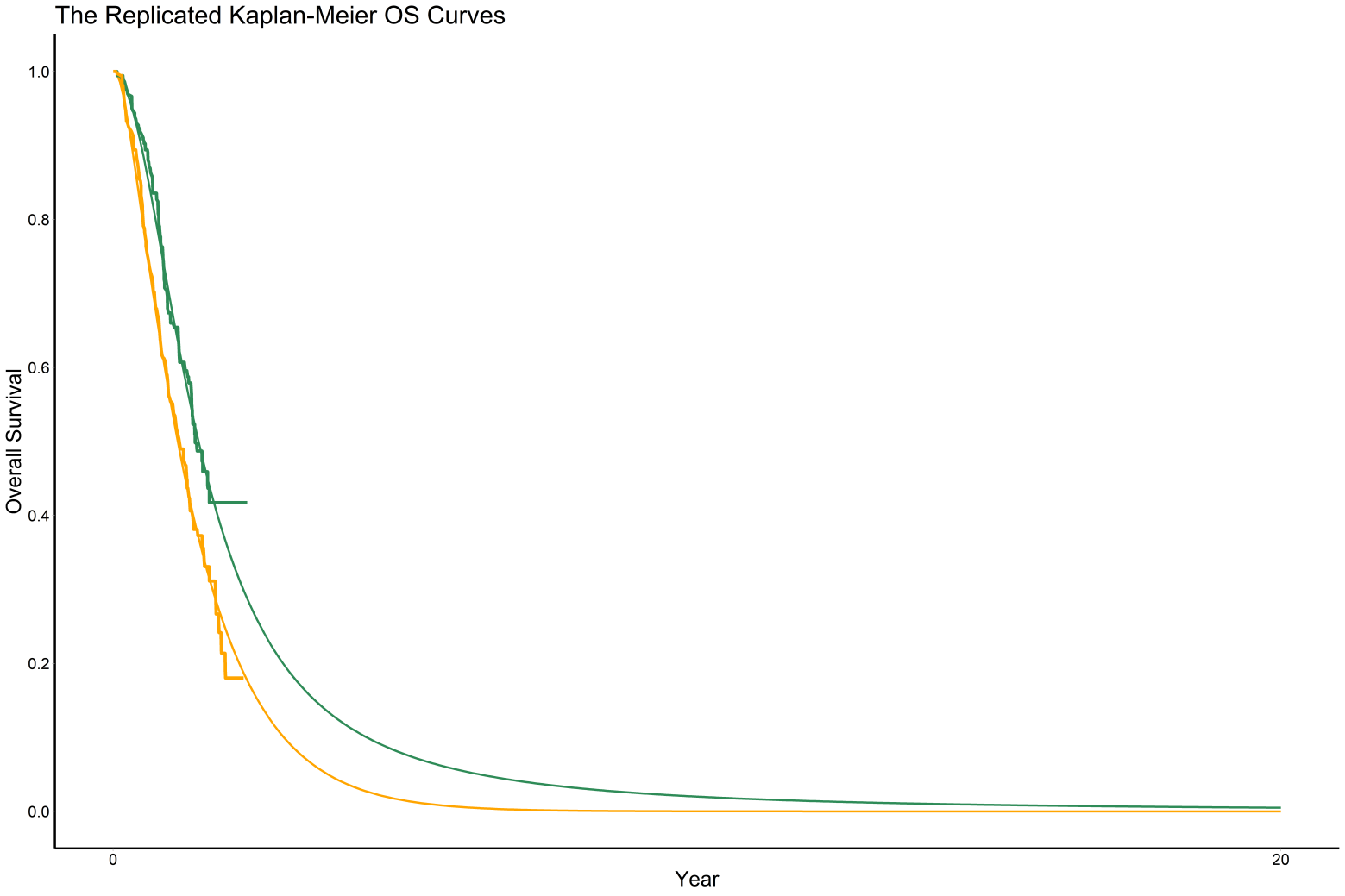
**

**Supplementary Figure 3 The Replicated Kaplan-Meier OS Curves in subgroup in patients with PD-L1 expression CPS ≥ 10**

### Supplementary Figure 4

**
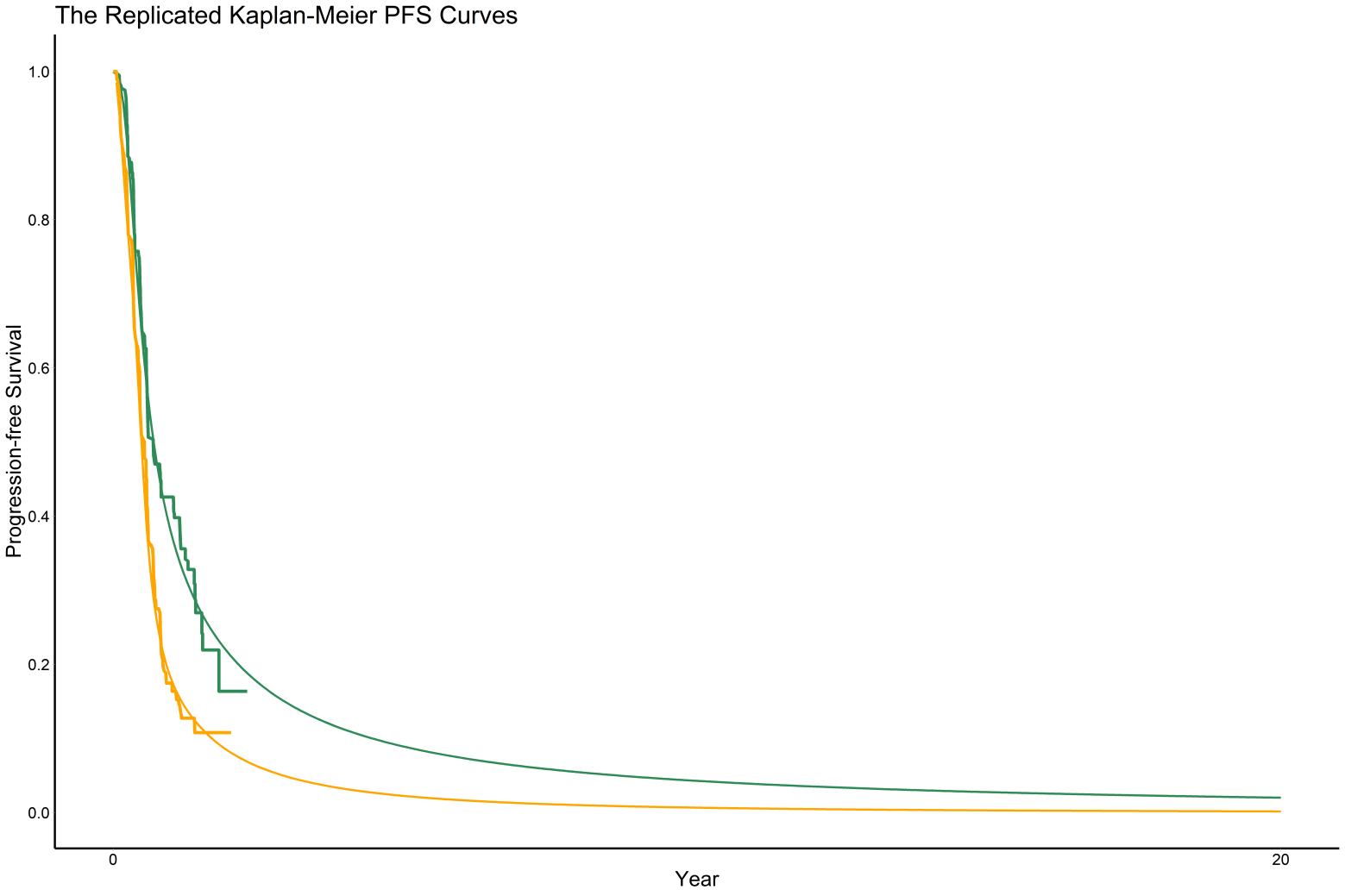
**

**Supplementary Figure 4 The Replicated Kaplan-Meier PFS Curves in subgroup in patients with PD-L1 expression CPS ≥ 10**

### Supplementary Figure 5

**
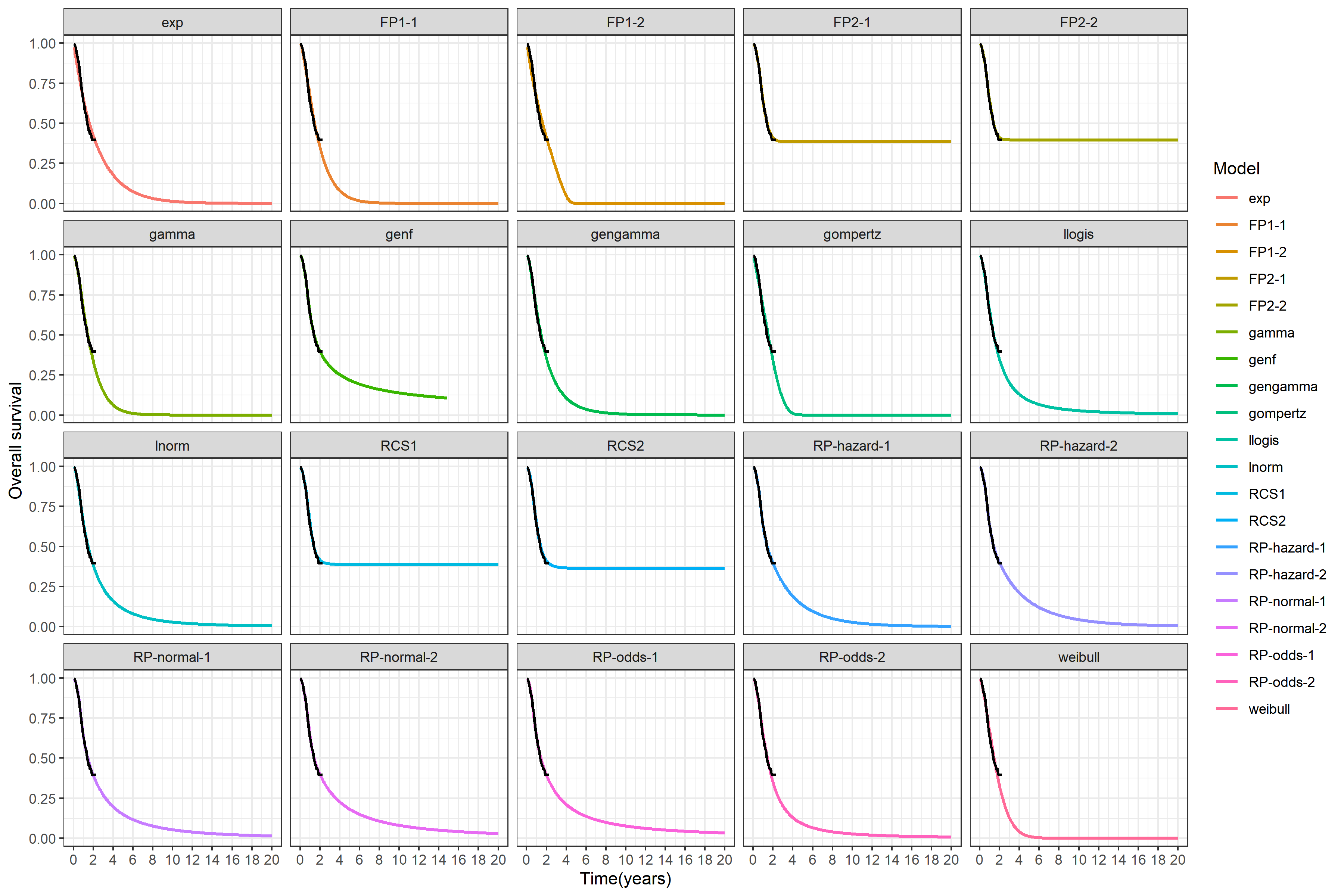
**

**Supplementary Figure 5 Extrapolation plot of different models for overall survival in sintilimab plus chemotherapy**

### Supplementary Figure 6

**
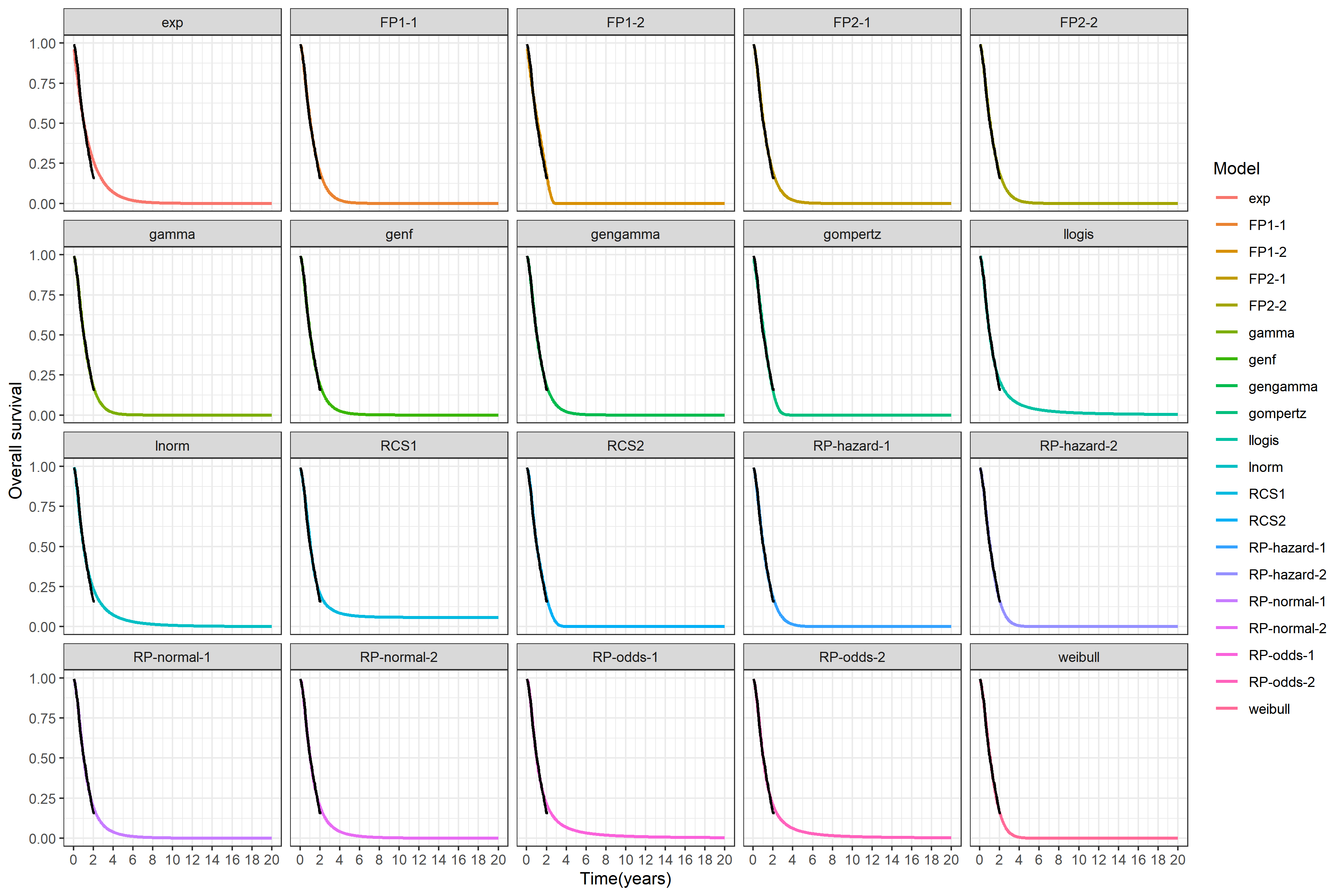
**

**Supplementary Figure 6 Extrapolation plot of different models for overall survival in chemotherapy**

### Supplementary Figure 7

**
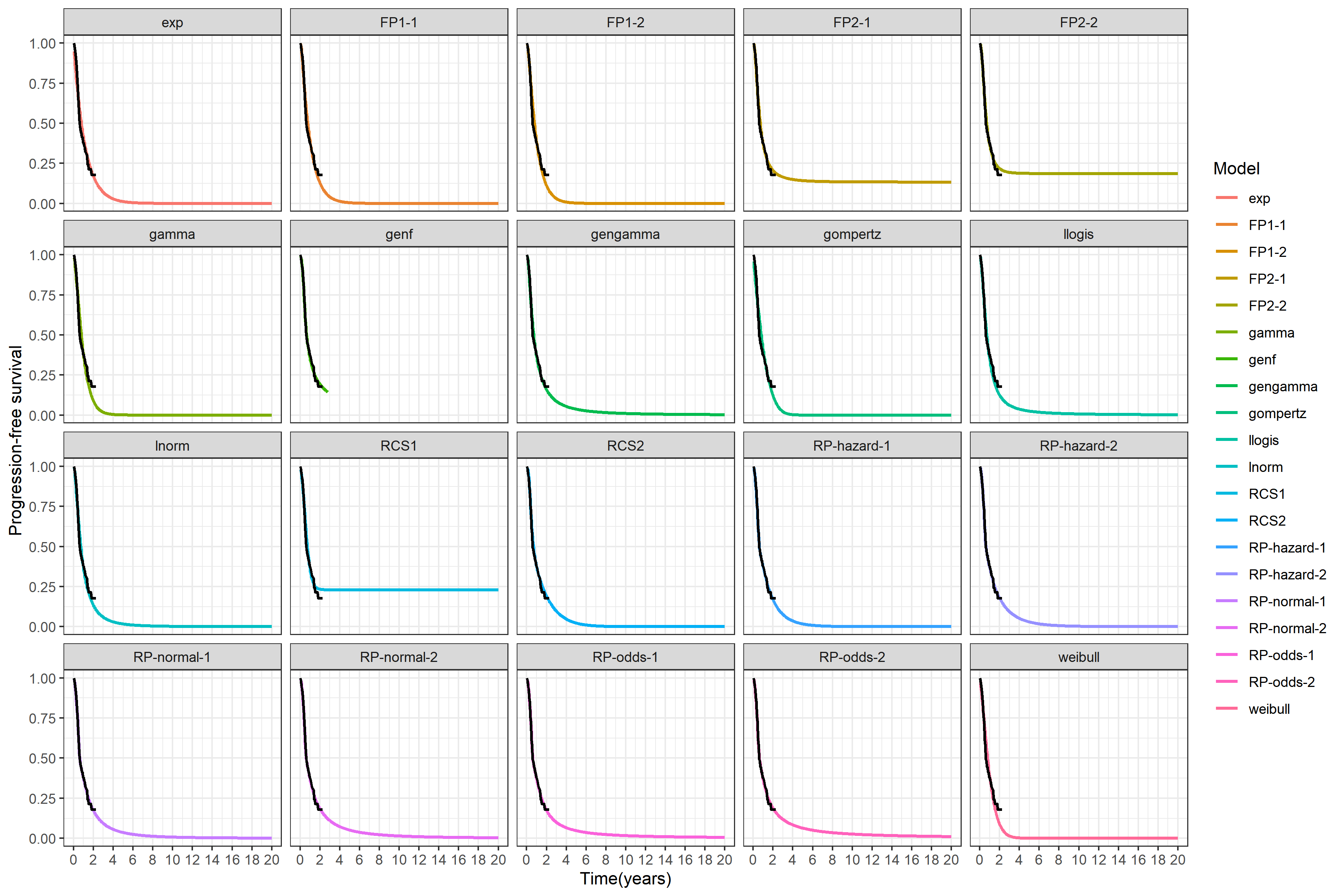
**

**Supplementary Figure 7 Extrapolation plot of different models for progression-free survival in sintilimab plus chemotherapy**

### Supplementary Figure 8

**
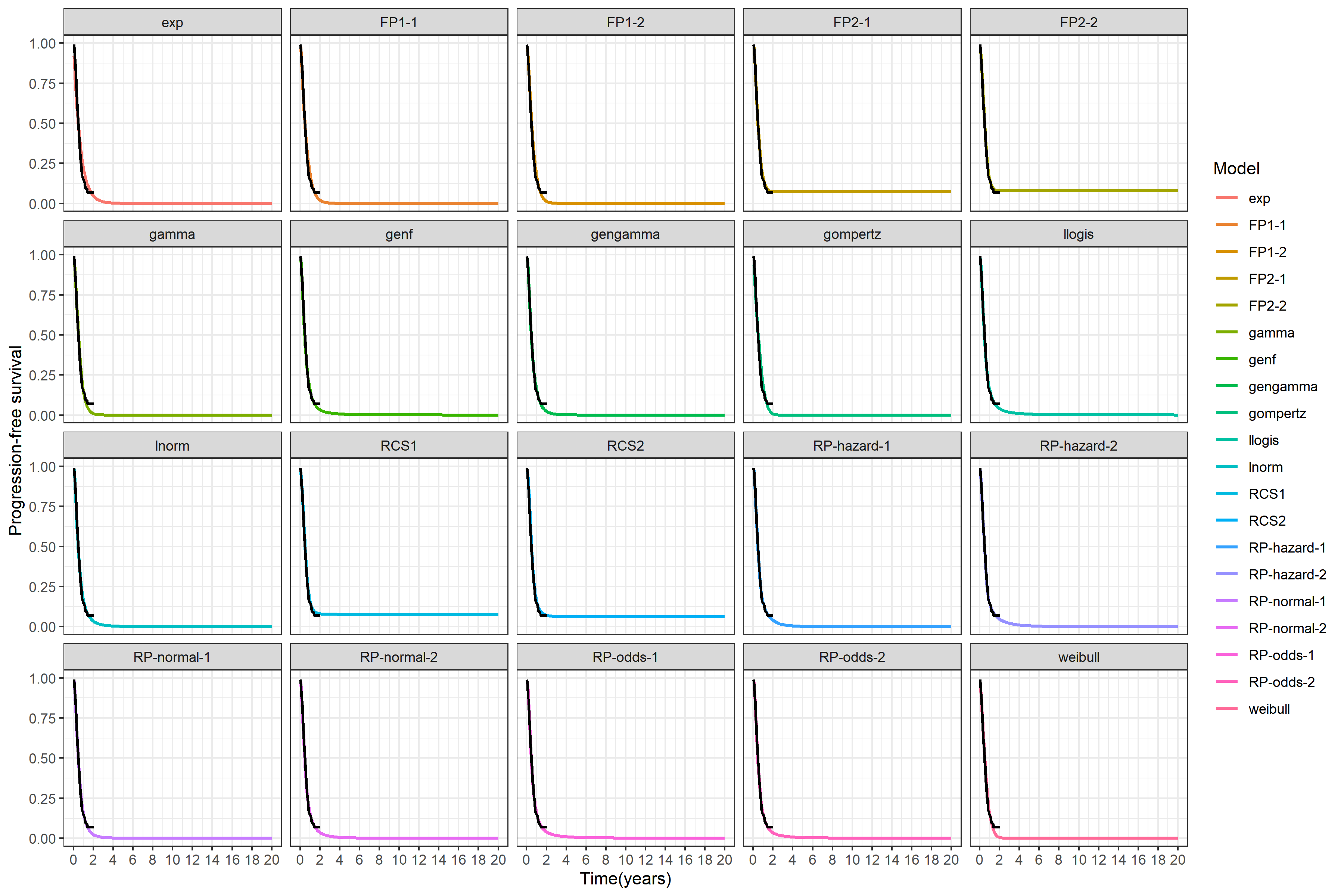
**

**Supplementary Figure 8 Extrapolation plot of different models for progression-free survival in chemotherapy**

### Supplementary Figure 9

**
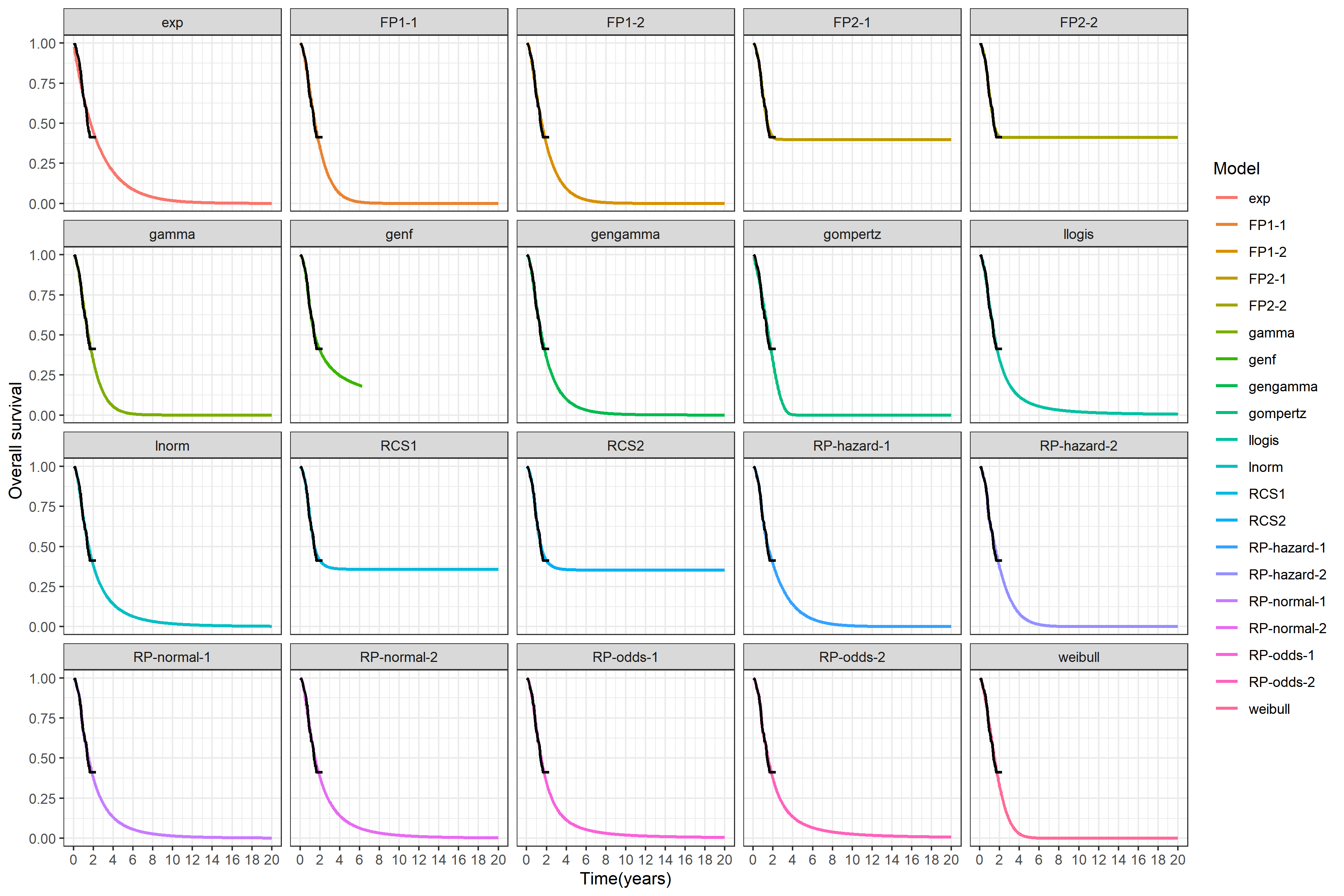
**

**Supplementary Figure 9 Extrapolation plot of different models for overall survival in sintilimab plus chemotherapy in patients with PD-L1 expression CPS ≥ 10**

### Supplementary Figure 10

**
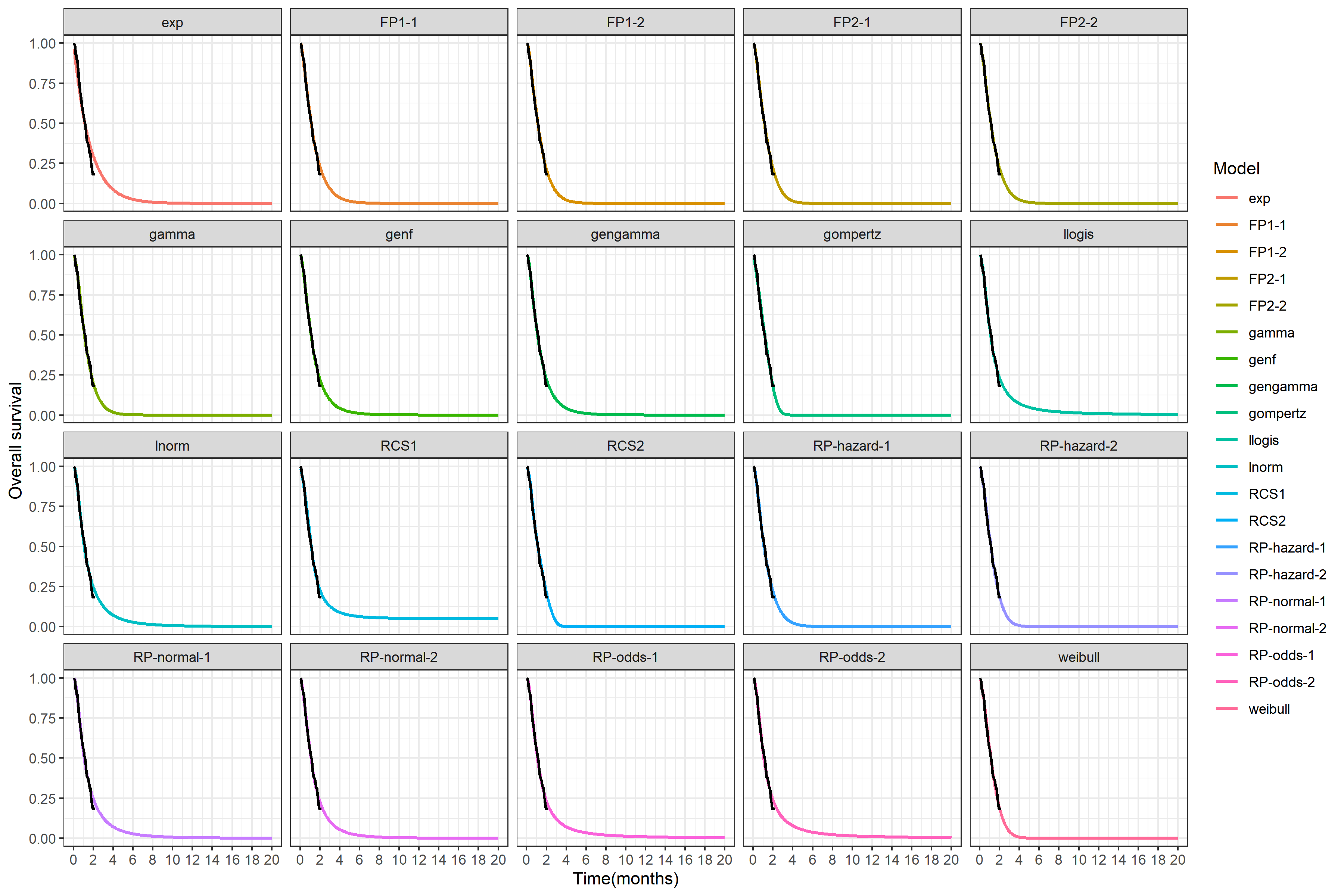
**

**Supplementary Figure 10 Extrapolation plot of different models for overall survival in chemotherapy in patients with PD-L1 expression CPS ≥ 10**

### Supplementary Figure 11

**
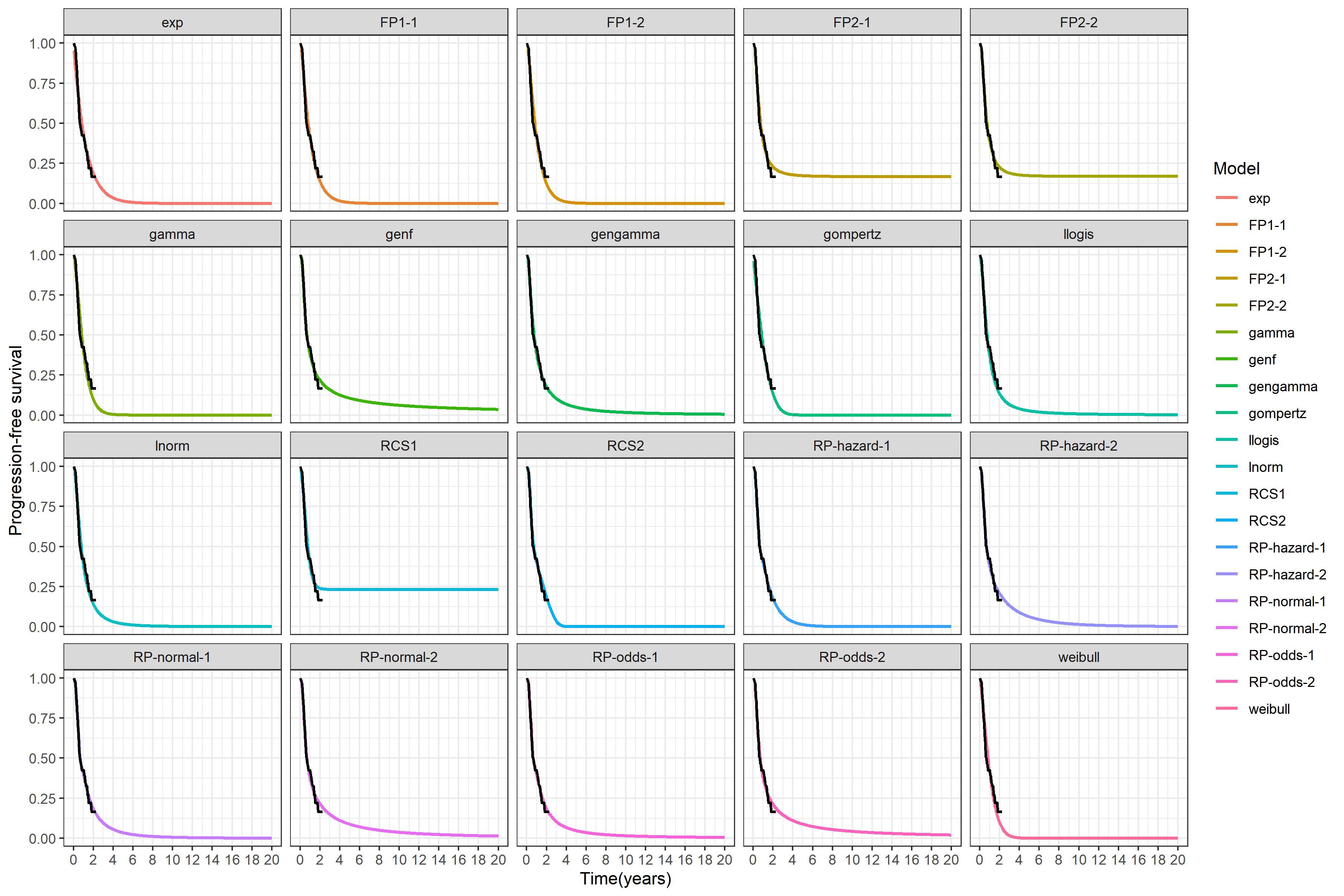
**

**Supplementary Figure 11 Extrapolation plot of different models for progression-free survival in sintilimab plus chemotherapy in patients with PD-L1 expression CPS ≥ 10**

### Supplementary Figure 12

**
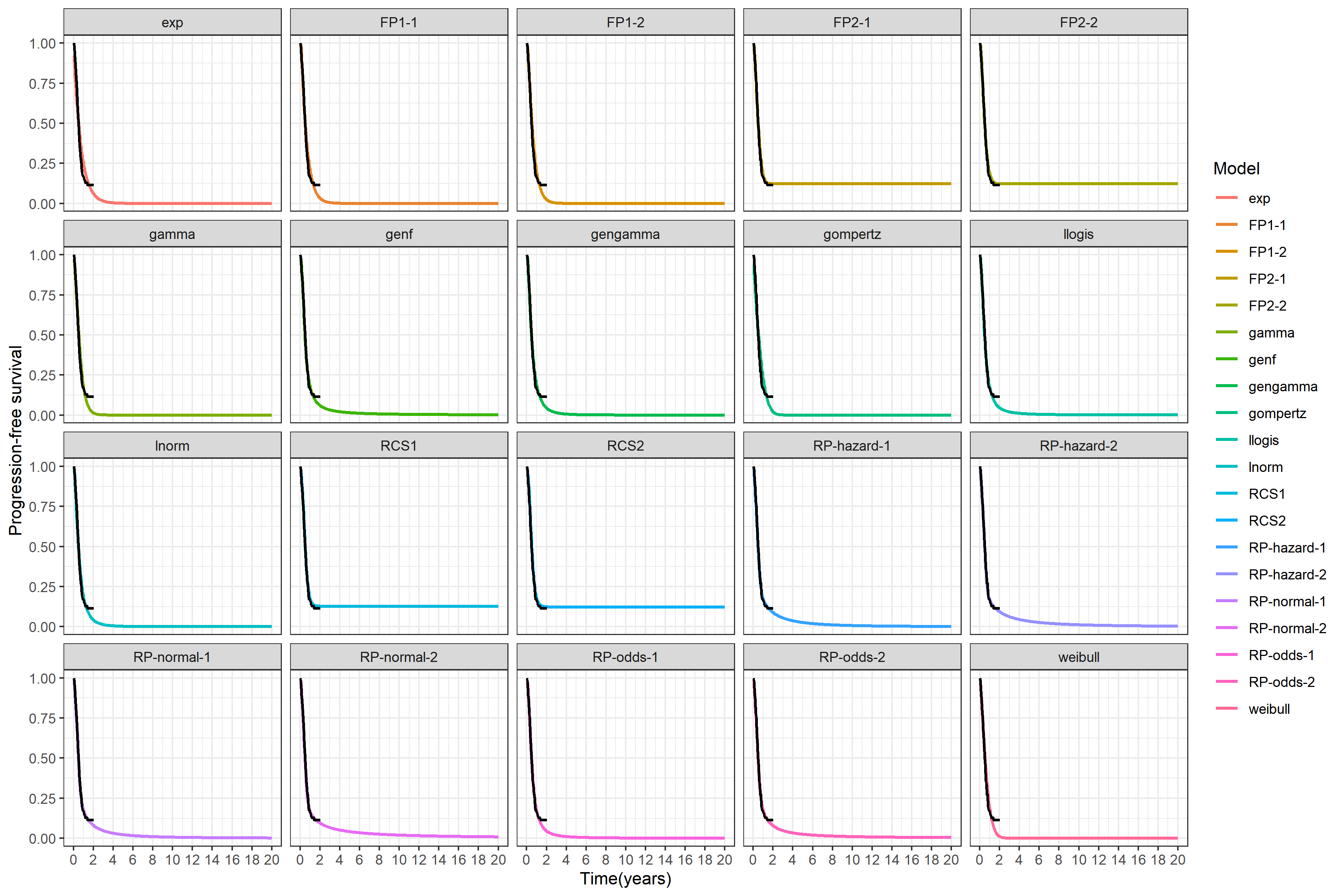
**

**Supplementary Figure 12 Extrapolation plot of different models for progression-free survival in chemotherapy in patients with PD-L1 expression CPS ≥ 10**

### Supplementary Figure 13

**
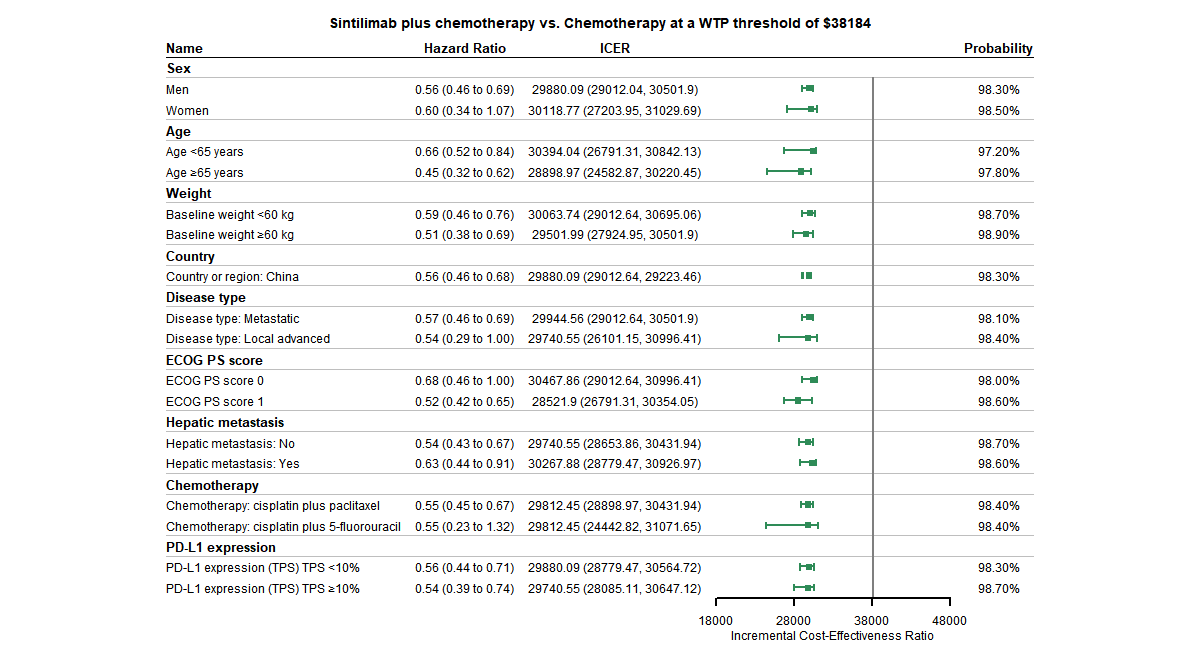
**

**Supplementary Figure 13 Subgroup Analysis Results of Incremental Cost-effectiveness Ratios (ICERs) and Probabilities of Cost-effectiveness Obtained by Varying the Hazard Ratios (HRs) for Progression Free Survival**
